# Age-related differences in circulating heparanase-1 activity in sepsis and correlation with systemic vascular endotheliopathy

**DOI:** 10.3389/fmed.2026.1893157

**Published:** 2026-08-12

**Authors:** Colin J. Sallee, Aaron S. Noa, Mouli Bhowmik, Amber C. Nobles, Zhangjie Wang, Jian Liu, Jillian R. Richter, J. Edwin Blalock, Rakesh P. Patel, Amit Gaggar, Robert P. Richter

**Affiliations:** 1 Division of Pediatric Critical Care Medicine, Department of Pediatrics, David Geffen School of Medicine, University of California, Los Angeles, CA, United States; 2 Heersink School of Medicine, University of Alabama at Birmingham, Birmingham, AL, United States; 3 Division of Pediatric Critical Care Medicine, Department of Pediatrics, Heersink School of Medicine, University of Alabama at Birmingham, Birmingham, AL, United States; 4 Division of Pulmonary, Allergy, and Critical Care Medicine, Department of Medicine, Heersink School of Medicine, University of Alabama at Birmingham, Birmingham, AL, United States; 5 Glycan Therapeutics, Raleigh, NC, United States; 6 Division of Chemical Biology and Medicinal Chemistry, Eshelman School of Pharmacy, University of North Carolina at Chapel Hill, Chapel Hill, NC, United States; 7 Division of Trauma and Acute Care Surgery, Department of Surgery, Heersink School of Medicine, University of Alabama at Birmingham, Birmingham, AL, United States; 8 Department of Pathology, Heersink School of Medicine, University of Alabama at Birmingham, Birmingham, AL, United States

**Keywords:** adults, children, endothelial glycocalyx, heparan sulfate, heparanase activity, sepsis, vascular endotheliopathy

## Abstract

**Background:**

Heparanase-1 (HPSE)-mediated degradation of endothelial glycocalyx heparan sulfate (HS) contributes to vascular endotheliopathy in sepsis, yet age-dependent differences in HPSE biology remain undefined. Thus, we sought to determine age-related differences in circulating HPSE activity during sepsis and its association with markers of endotheliopathy and organ dysfunction.

**Methods:**

Heparanase-1 enzymatic activity and HS disaccharide levels were measured in plasma from children (10 sepsis, 10 controls) and adults (16 sepsis, 15 controls) from prospective observational cohorts using liquid chromatography-tandem mass spectrometry. Associations with plasma angiopoietin-2 levels and change in serum albumin (markers of endotheliopathy) in addition to organ failure scores were assessed using Spearman correlations.

**Results:**

Heparanase-1 activity and circulating HS were elevated in both sepsis cohorts compared to controls, with moderate-to-strong correlations between HPSE activity and HS levels. However, adults with sepsis demonstrated approximately 10-fold higher plasma HPSE activity than children (median 1,256 vs. 116, *p* < 0.001), despite children exhibiting greater endotheliopathy and higher organ failure scores. In both age groups, HPSE activity correlated with angiopoietin-2, serum albumin decline, and organ failure scores. Adult non-survivors had higher HPSE activity than survivors; no pediatric deaths occurred. Predominant neutrophilic/monocytic activation in adults versus greater platelet consumption in children may suggest developmental differences in cellular sources of circulating HPSE.

**Conclusion:**

Heparanase-1-mediated glycocalyx degradation is a conserved feature of sepsis across the age spectrum, but the magnitude, cellular source, and clinical implications of circulating HPSE activity differ markedly by age, underscoring the need for age-stratified therapeutic approaches.

## 1 Introduction

Sepsis remains a leading cause of mortality and life-altering morbidity globally, disproportionately impacting individuals at the extremes of age (1). Although sepsis pathobiology is complex and incompletely understood, systemic vascular endotheliopathy–characterized by pathological endothelial cell (EC) activation and degradation of the luminal endothelial glycocalyx–is a unifying feature across sepsis phenotypes that promotes vascular dysfunction and downstream organ injury (2). Identifying principal drivers of vascular endotheliopathy during acute sepsis may thus reveal novel therapeutic targets.

Schmidt et al. (3) demonstrated that heparanase-1 (HPSE) may be a key mediator of vascular endotheliopathy in the lungs of adults with sepsis through degradation of heparan sulfate (HS) within the endothelial glycocalyx. Subsequent studies have shown that HPSE contributes to other sepsis-related organ injuries, but these investigations focused on adults or adult-aged animal models. We have previously observed increased levels of HPSE activity in the circulation of children with sepsis and demonstrated that enzymatic degradation of HS from the glycocalyx of cultured human microvascular ECs drives a pro-inflammatory, pro-angiogenic phenotype (4, 5). We also previously showed that levels of circulating HS fragments rise concurrently with plasma interleukin-6 levels and that elevations in circulating HS levels occur prior to the rise in circulating levels of angiopoietin-2, a biomarker of pathological endothelial activation (4), in a murine sepsis model. Collectively, these findings support circulating HPSE as a mechanistic contributor to systemic vascular endotheliopathy in sepsis. However, the role of HPSE in the pathogenesis of sepsis-related vascular endotheliopathy across the age spectrum remains unknown.

We therefore sought to quantify the enzymatic activity of HPSE in the circulation of contemporary pediatric and adult sepsis cohorts as enzyme activity is more reflective of the pathobiology occurring during sepsis. We also sought to determine the association of circulating HPSE activity with biochemical and clinical measures of endothelial injury and organ dysfunction and define age-dependent differences in HPSE biology that may contribute to heterogeneous clinical outcomes. As prior work suggests that the innate immune response is more robust in children with sepsis compared to adults (6), we hypothesized that circulating HPSE activity levels are more significantly elevated in children with sepsis compared to adults. Moreover, as we have previously shown that circulating levels of HS correlate with angiopoietin-2 in children with sepsis (4) and vascular endotheliopathy is a shared pathological feature of sepsis in both children and adult populations (7, 8), we also hypothesized that circulating levels of HPSE activity are associated with markers of vascular endotheliopathy and organ dysfunction in both age groups.

## 2 Materials and methods

### 2.1 Human subjects and study design

We leveraged access to plasma samples from 16 adults with sepsis and 15 healthy adult controls that were collected for a separate prospective observational study performed between August 2021 and July 2023. We also used available plasma samples from 10 children with sepsis and 10 healthy controls who were recruited for a separate prospective observational study performed between March 2023 and June 2025. Adults with sepsis were enrolled within 24 h of hospitalization at the University of Alabama Hospital. Previously healthy adult controls were recruited from the University of Alabama at Birmingham Heersink School of Medicine. Children with sepsis were enrolled within 24 h of hospitalization at Children’s of Alabama Hospital. Sepsis was diagnosed in adult subjects using the Sepsis-3 criteria (9); sepsis was diagnosed in pediatric subjects using the International Pediatric Sepsis Consensus Conference definition for sepsis (10) prior to February 2024 and the International Consensus Criteria for Pediatric Sepsis and Septic Shock (Phoenix criteria) (11) after February 2024 when these clinical criteria were introduced to the pediatric critical care community (importantly, subjects enrolled prior to February 2024 also met Phoenix criteria for pediatric sepsis). Previously healthy children presenting to the Children’s of Alabama operating room for dental cleaning procedures were enrolled as healthy controls. The study was approved by the UAB Institutional Review Board (protocols 300001022, 300005209).

### 2.2 Sample collection

Whole blood samples were collected into sodium citrate vacutainers from pediatric and adult subjects with sepsis within 12 and 24 h of hospitalization, respectively. Platelet poor plasma was isolated by double centrifugation (1,500 × *g* for 15 min) of sodium citrated whole blood and stored at −80 °C. Pediatric samples had sufficient volume for all assays. Because adult subjects with sepsis and 10 adult controls had insufficient plasma volume available for all assays, equal volumes of plasma from 2 adult subjects (control group matched by age; sepsis group matched by age and APACHE II scores) within each group were pooled, generating 8 adult sepsis samples and 10 adult control samples for analyses. Importantly, sensitivity analysis demonstrated no significant difference in HPSE enzymatic activity between the 5 unpooled and 5 pooled samples from adult controls [unpooled group, 58 U/mL (IQR 21, 121) vs. pooled group, 137 U/mL (IQR 40, 261), *p* = 0.198], suggesting an expected averaging effect of the pooling process.

### 2.3 Clinical data

Demographic and clinical data were extracted from subjects’ electronic health records and recorded. Organ dysfunction during the first 24 h of hospitalization was quantified using Sequential Organ Failure Assessment (SOFA) scores for adults (9) and Pediatric Sequential Organ Failure Assessment (pSOFA) scores for children (12). Organ failure scores were presumed to be 0 for controls. For pooled adult samples, clinical values were averaged between paired subjects.

### 2.4 HPSE activity and HS disaccharide measurement

HPSE enzymatic activity was measured in 20 μL plasma using our liquid chromatography-tandem mass spectrometry (LC-MS/MS)-based method (13). HS disaccharides were quantified from 200 μL plasma using LC-MS/MS as we have previously (5, 14). No participants received unfractionated or low-molecular-weight heparin, which are known inhibitors of HPSE enzymatic activity (15), at the time of sampling.

### 2.5 Biomarkers of vascular endotheliopathy

Angiopoietin-2 levels were measured by enzyme-linked immunosorbent assay (cat#D&623, R&D Systems). Change in serum albumin from admission to 24 h served as a surrogate marker of vascular permeability (presumed to be 0 for controls) (16).

### 2.6 Cell isolation from whole blood

Neutrophils, monocytes, and platelets were isolated from a healthy adult male donor using techniques described previously that generate a high purity of respective cell populations (17–20). Briefly, 40 mL of whole blood were collected into K2-EDTA blood collection tubes for neutrophil isolation, 48 mL of whole blood were collected into CPT blood collection tubes for monocyte isolation, and 45 mL of whole blood were collected into 3.2% sodium citrate blood collection tubes for platelet isolation. The blood designated for neutrophil isolation was mixed with 40 mL 3% dextran 0.9% NaCl solution and incubated for 20 min at room temperature to allow cellular fraction settling. After removing the plasma supernatant, the concentrated cell suspension was centrifuged at 250 × *g* for 10 min at room temperature. The cell pellet was resuspended in 20 mL 0.9% NaCl. Ten milliliters of Ficoll-Paque PLUS was instilled below the cell suspension, and the cell suspension-Ficoll solution was centrifuged at 400 × *g* for 40 min at room temperature. The cell pellet was resuspended in 20 mL cold 0.2% NaCl solution, 20 mL cold 1.6% NaCl solution was added to lyse residual erythrocytes, and the cell suspension was centrifuged at 250 × *g* for 6 min at 4 °C. The resulting neutrophil cell pellet was resuspended in 1 mL 1× phosphate buffered saline without calcium or magnesium [PBS(−/−)] and centrifuged at 250 × *g* for 6 min at 4 °C twice to wash the cells. The remaining cell pellet was resuspended in 100 μL mammalian protein extraction reagent (M-PER; cat#78501, Thermo Scientific), 1:100 v/v protease/phosphatase inhibitor (cat#78415, Thermo Scientific, and cat#5246251SET, MilliporeSigma), incubated at −80 °C for 1 h, and thawed to lyse the neutrophils. Cell lysate was centrifuged at 18,213 × *g* for 20 min at 4 °C, and the supernatant was collected and stored at −80°C.

To isolate monocytes, CPT tubes were centrifuged at 1,500 × *g* for 20 min at room temperature. After removing 3 mL of plasma from the top of each CPT tube, the remaining 4.5 mL of plasma and peripheral blood mononuclear cell (PBMC) suspension layered at the top of each CPT above the gel plug were transferred to sterile conical tubes and centrifuged at 300 × *g* for 10 min at room temperature. After resuspending PBMCs in 1× PBS(−/−) and re-centrifuging at 300 × *g* for 10 min at room temperature, the PBMC cell pellet was resuspended in 1 mL RPMI 1640 culture medium supplemented with 10% fetal bovine serum and 1% penicillin-streptomycin. PBMCs were transferred to a sterile tissue culture plate and incubated at 37 °C in a humidified environment supplemented with 5% CO_2_ for 24 h. Non-adherent cells (lymphocytes) were removed with the culture medium, and adherent cells (monocytes) were washed twice with 1× PBS(−/−).(21) Monocytes were then lysed with 60 μL M-PER, 1:100 v/v protease/phosphatase inhibitor cocktail on ice for 10 min. Cell lysate was harvested and centrifuged at 18,213 × *g* for 20 min at 4 °C, and the supernatant was collected and stored at −80°C.

To isolate platelets, citrated whole blood was centrifuged at 200 × *g* for 20 min at room temperature. Platelet-rich plasma (PRP) was transferred to a separate, sterile conical tube, and an equal volume of 140 mM NaCl, 2.7 mM KCl, 3.8 mM HEPES, 5 mM EGTA, 1 μM PGE_1_ buffer was added. The PRP-buffer admixture was centrifuged at 200 × *g* for 20 min at room temperature, and the supernatant was transferred to a separate, sterile conical tube and centrifuged at 800 × *g* for 20 min at room temperature. The resulting platelet pellet was washed twice with 150 mM NaCl, 10 mM sodium citrate, 1 mM EDTA, 1% dextrose, 1 μM PGE_1_ buffer and resuspended in 100 μL M-PER, 1:100 v/v protease/phosphatase inhibitor. After 1 h of incubation at −80 °C, the platelet lysate was centrifuged at 18,213 × *g* for 20 min at 4 °C, and the supernatant was harvested and stored at −80°C.

### 2.7 Cell culture

Primary human lung microvascular ECs (HLMVEC) (cat#540-05a, Cell Applications; obtained from the vendor at passage 3) from an adult human female (per vendor) were cultured as we have previously (4). In brief, 5 × 10^5^ HLMVECs (passage 4) per well were seeded into 3 wells of a 6-well tissue culture plate pre-incubated with attachment factor solution (cat#123-500, Cell Applications) and grown to 100% confluence. The HLMVEC monolayers were washed twice with 1× PBS(−/−) and incubated with 60 μL M-PER, 1:100 v/v protease/phosphatase inhibitor for 20 min on ice. Cell lysate from the 3 wells was combined and centrifuged at 18,213 × g for 20 min at 4 °C. Cell lysate supernatant was collected and stored at −80°C.

### 2.8 Western blot

Cell lysates (20 μg) were resolved by gel electrophoresis alongside 20 ng recombinant, active human HPSE protein (cat#7570-GH-005, R&D Systems) as a positive control, transferred to a nitrocellulose membrane, blocked for 1 h, and probed using rabbit monoclonal antibody against human HPSE (1:500; cat#ab288438, Abcam) and glyceraldehyde 3-phosphate dehydrogenase (1:1,000; cat#2118S, Cell Signaling Technology) for 16 h at 4 °C. The membrane was incubated with horseradish peroxidase (HRP)-conjugated TidyBlot western blot detection reagent (1:5,000; cat#STAR209PA, Bio-Rad) for 1 h. HRP substrate (cat#WBLUF0100, MilliporeSigma) was applied to the membrane, and immunoreactive bands were detected using an Amersham ImageQuant 800 Fluor imager (Cytiva).

### 2.9 Statistical analysis

Categorical variables are presented as number (percent). Continuous variables are presented as median (interquartile range, or IQR). Comparisons between 2 continuous variables were performed using the Wilcoxon rank-sum test, and comparisons of continuous variables across 3 or more groups were performed using the Kruskal-Wallis 1-way analysis of variance followed by Dunn’s multiple comparisons test. Probability (p) values of multiple comparisons tests were adjusted using the 2-stage step-up method of Benjamini, Krieger, and Yekutieli to control the false discovery rate. Correlations were assessed with Spearman’s rank test. Analyses were performed with and without statistical outliers (>1.5× IQR above the third quartile or below the first quartile); outliers were excluded if they significantly altered results (see Supplementary Table 1 for analyses with and without outliers). All comparisons were 2-sided (α = 0.05), and *p*-values < 0.05 were considered significant. Analyses were performed using GraphPad Prism (v10.4.2).

## 3 Results

### 3.1 Characterization of HPSE enzymatic activity in plasma of children and adults with sepsis

We analyzed plasma samples from 16 adults with sepsis [median 62 (IQR 48,67) years old; 38% male] and 15 healthy adult controls [40 (IQR 46,52) years old; 25% male] prospectively collected between August 2021 and July 2023 and plasma samples from 10 children with sepsis [median 5 (IQR 2,10) years old; 70% male] and 10 healthy controls [5 (IQR 4,6) years old; 50% male] prospectively collected between March 2023 and June 2025. Subject demographics are summarized in Table 1.

**TABLE 1:** Demographic and clinical data for children and adults with sepsis and respective controls.

Variable	Pediatric control (*n* = 10)	Pediatric sepsis (*n* = 10)	Adult control (*n* = 15)	Adult sepsis (*n* = 16)
Demographic data				
Age, years	5 (4, 5.5)	4.5 (2, 10)	40 (46, 52)^g,h^	62 (48, 67)^k,l^
Male sex, *n* (%)	5 (50)	7 (70)	4 (25)	6 (38)
Comorbidities				
Cardiovascular	0 (0)	3 (30)	0 (0)	10 (63)
Respiratory	0 (0)	3 (30)	0 (0)	4 (25)
Renal	0 (0)	3 (30)	0 (0)	3 (19)
Neurologic	0 (0)	1 (10)	0 (0)	2 (13)
Hematologic/oncologic	0 (0)	1 (10)	0 (0)	2 (13)
Endocrine	0 (0)	1 (10)	0 (0)	5 (31)
Hepatic/gastrointestinal	0 (0)	4 (40)	0 (0)	1 (6)
Rheumatologic	0 (0)	0 (0)	0 (0)	2 (13)
Clinical data				
Infection source				
Respiratory	–	5 (50)	–	6 (38)
Central venous catheter	–	2 (2)	–	1 (6)
Skin/soft tissue	–	1 (10)	–	2 (13)
Gastrointestinal	–	1 (10)	–	2 (13)
Urinary tract	–	1 (10)	–	3 (19)
Indeterminate	–	0 (0)	–	1 (6)
Absolute neutrophil count (×10^3^/μL), admission^a^	–	12 (5.9, 34.1)	–	12.4 (8.5, 27.3)
Absolute lymphocyte count (×10^3^/μL), admission^a^	–	0.9 (0.6, 1.6)	–	0.6 (0.3, 1.1)
Absolute monocyte count (×10^3^/μL), admission^a^	–	0.2 (0.1, 0.3)	–	0.6 (0.3, 1.7)^m^
Platelet count (×10^3^/μL), admission^a^	–	214 (56, 263)	–	182 (109, 235)
Serum albumin (g/dL), admission^a^	–	3.1 (2.7, 3.9)	–	2.8 (2.6, 3.2)
Angiopoietin-2 level (ng/mL), admission	0.46 (0.52, 0.63)	4.48 (3.05, 6.12)^d^	0.33 (0.23, 0.42)^i^	1.79 (1.70, 2.27)^n^
PELOD-2 score, initial^c^	0 (−)	10 (7, 13)^e^	–	–
APACHE II score^c^	–	–	0 (−)	12 (11,17)^e^
Corticosteroid use	0 (0)	4 (40)	0 (0)	8 (50)
Vasopressor use	0 (0)	9 (90)	0 (0)	12 (75)
Maximum respective organ failure assessment score, first 24 h^b,c^	0 (−)	13 (10, 17)^f^	0 (−)^j^	6 (5, 8)^k,o^
28-day mortality, *n* (%)	0 (0)	0 (0)	0 (0)	5 (31)^p^

Data presented as median (interquartile range) or number (percentage).

^a^Clinical laboratory values unavailable for pediatric and adult controls.

^b^Pediatric Sequential Organ Failure Assessment scores were used for pediatric subjects, and sepsis-related organ failure assessment scores were used for adult subjects.

^c^Organ failure assessment/severity of illness scores were determined to be 0 for pediatric and adult control subjects.

^d^*p* = 0.002, Kruskal-Wallis 1-way ANOVA followed by Dunn’s multiple comparisons test: pediatric sepsis vs. pediatric control.

^e^*p* < 0.001, Wilcoxon rank sum test.

^f^*p* < 0.001, Kruskal-Wallis 1-way ANOVA followed by Dunn’s multiple comparisons test: pediatric sepsis vs. pediatric control.

^g^*p* = 0.002, Kruskal-Wallis 1-way ANOVA followed by Dunn’s multiple comparisons test: adult control vs. pediatric control.

^h^*p* = 0.004, Kruskal-Wallis 1-way ANOVA followed by Dunn’s multiple comparisons test: adult control vs. pediatric sepsis.

^i^*p* = 0.002, Kruskal-Wallis 1-way ANOVA followed by Dunn’s multiple comparisons test: adult control vs. pediatric sepsis.

^j^*p* < 0.001, Kruskal-Wallis 1-way ANOVA followed by Dunn’s multiple comparisons test: adult control vs. pediatric sepsis.

^k^*p* < 0.001, Kruskal-Wallis 1-way ANOVA followed by Dunn’s multiple comparisons test: adult sepsis vs. pediatric control.

^l^*p* < 0.001, Kruskal-Wallis 1-way ANOVA followed by Dunn’s multiple comparisons test: adult sepsis vs. pediatric sepsis.

^m^*p* = 0.015, Wilcoxon rank sum test.

^n^*p* = 0.004, Kruskal-Wallis 1-way ANOVA followed by Dunn’s multiple comparisons test: adult sepsis vs. adult control.

^o^*p* < 0.001, Kruskal-Wallis 1-way ANOVA followed by Dunn’s multiple comparisons test: adult sepsis vs. adult control.

^p^*p* = 0.004, Fisher’s exact test. APACHE II represents Acute Physiology and Chronic Health Evaluation; PELOD-2, Pediatric Logistic Organ Dysfunction-2.

**FIGURE 1:**
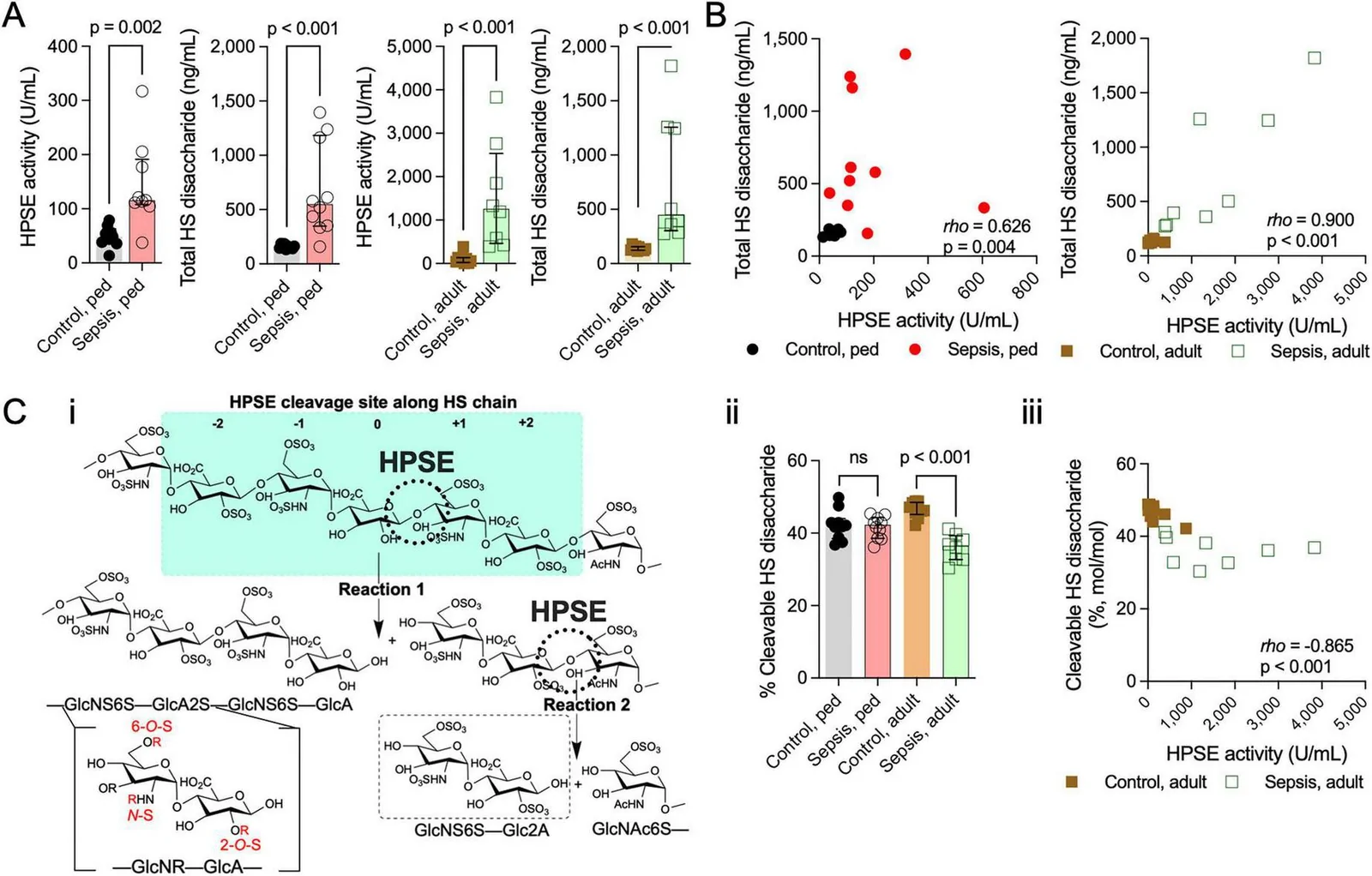
Relationship between plasma HPSE activity levels and markers of vascular disease, measures of organ injury, and clinical outcomes. **(A)** HPSE activity and HS levels in children with sepsis (*n* = 10) and adults with sepsis (*n* = 8) relative to controls (pediatric control, *n* = 9; adult control, *n* = 9). **(B)** Spearman’s rank correlations between HPSE activity and HS levels the respective age groups. **(C)** (i) HPSE cleavage site along HS with two putative reactions that generate HS disaccharide fragments rich in 6-*O*-sulfation of the glucosamine residue with or without *N*-sulfation (GlcNS6S or GlcNAc6S) and 2-*O*-sulfation of the glucuronic acid residue (GlcA2S); inset depicts an HS disaccharide with candidate *N*-, 6-*O*-sulfation sites on glucosamine (GlcNR) and the 2-*O*-sulfation site of hexuronic acid (UA); (ii) percentage of plasma HS with “cleavable” sulfation motifs enriched in 6-*O*-, *N*-, and 2-*O*-sulfation (22, 23) in children and adults with and without sepsis; (iii) Spearman’s rank correlation between HPSE activity and percentage of HS with cleavable domains in adults. Data presented as median (IQR). Comparisons made using the Wilcoxon rank-sum test **(A)** or Kruskal-Wallis 1-way ANOVA **(Cii).**

Children and adults with sepsis had significantly higher levels of HPSE enzymatic activity and soluble HS in their circulation compared with controls (Figure 1A) with moderate-to-strong positive correlations noted between HPSE activity and HS levels in both age groups (Figure 1B), suggesting glycocalyx degradation as a shared feature of sepsis pathobiology. However, children with sepsis had significantly lower plasma HPSE activity compared to adults with sepsis [116 U/mL (IQR 109, 191) vs. 1,256 U/mL (IQR 465, 2,534), *p* < 0.001]. Notably, circulating levels of HPSE activity were similar between pediatric and adult controls [49 U/mL (IQR 37, 63) vs. 73 U/mL (IQR 24, 134), *p* = 0.258].

We then analyzed the biochemical composition of circulating HS chains to support the noted difference in circulating HPSE activity between children and adults with sepsis with the hypothesis that HS chains within plasma samples with higher HPSE enzymatic activity would contain HS with lower available cleavage sites for HPSE due to degradation by HPSE prior to plasma harvest. Peterson and Liu (22, 23) previously demonstrated that HPSE cleaves HS chains at highly sulfated regions of HS between a glucuronic acid (GlcA) residue and N-sulfated glucosamine residue (GlcNS) that are surrounded by glucuronic acid residues containing 2-O-sulfation (GlcA2S) (Figure 1Ci). Importantly, our method for analyzing the sulfation of disaccharides within intact HS chains in plasma first filters out HS di- and tetra-saccharide fragments that are cleaved from larger HS chains prior to plasma harvest (5, 14); thus, results from our HS disaccharide analysis only represent HS chains that are intact at the time of plasma collection. We found that the relative percentage of HS disaccharides within the HPSE cleavage site (e.g., GlcNS6S-GlcA2S, GlcNS-GlcA2S, GlcNAc6S-GlcA2S, see Figure 1Ci), or “cleavable” HS disaccharides, was significantly lower in adults with sepsis relative to controls but not in children with sepsis compared to controls (Figure 1Cii). Furthermore, the percentage of “cleavable” HS disaccharides in adults demonstrated a strong inverse correlation with HPSE activity (Figure 1Ciii), Together, these data suggest that adults with sepsis have significantly higher plasma HPSE activity on circulating HS chains prior to sample collection compared to children with sepsis. Interestingly, despite adults with sepsis having higher plasma HPSE activity, total circulating levels of HS were similar between the sepsis groups [children, 550 ng/mL (IQR 347, 1,182) vs. adults, 449 (IQR 304, 1,256), *p* > 0.99].

### 3.2 Associations between HPSE activity levels and markers of endotheliopathy and organ injury

**FIGURE 2:**
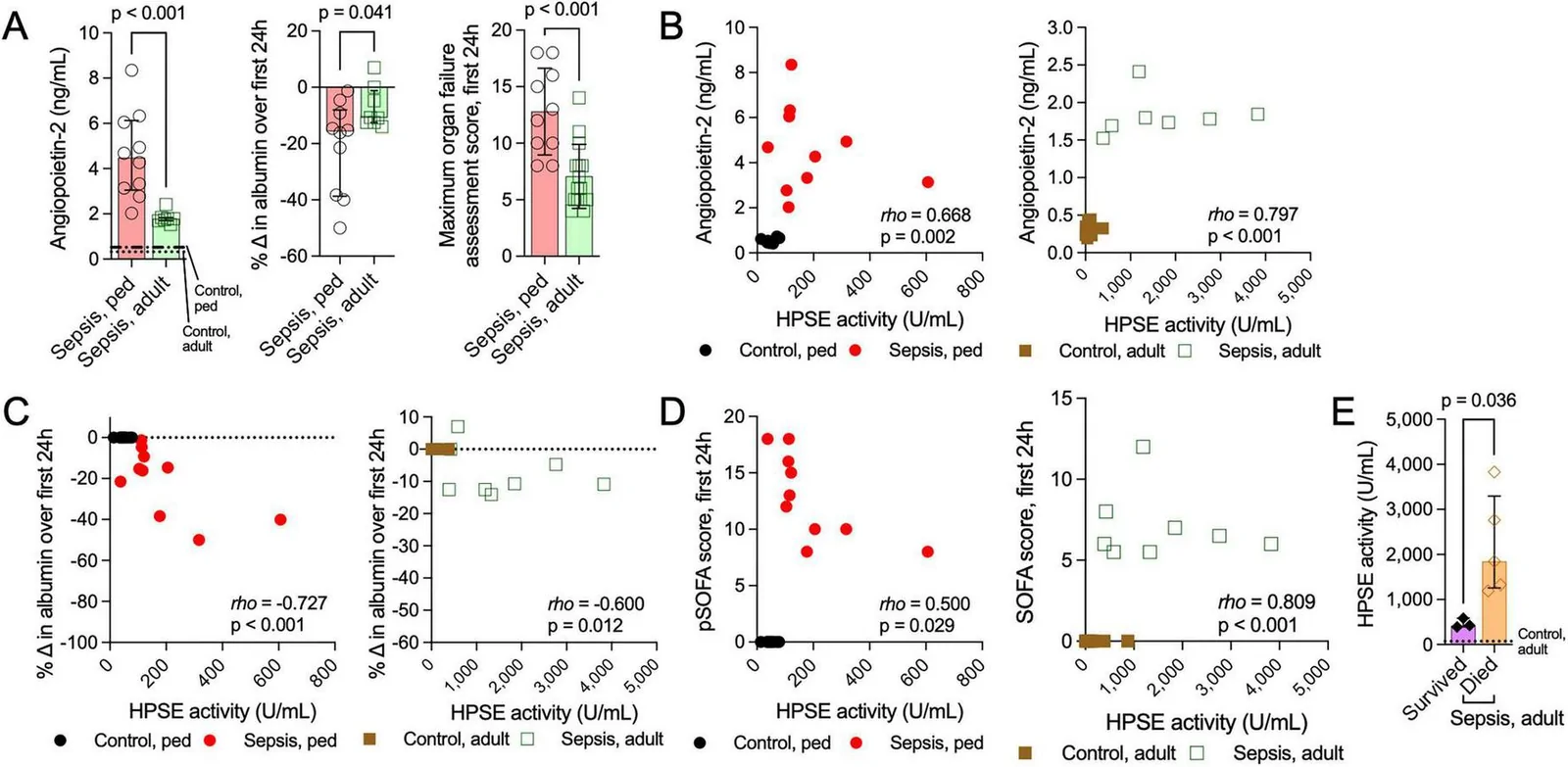
Relationship between plasma HPSE activity levels and markers of vascular disease, measures of organ injury, and clinical outcomes. **(A)** Plasma levels of angiopoietin-2 at admission, change in serum albumin levels over the first 24 h of hospitalization, and maximum organ failure assessment scores within the first 24 h of hospitalization in children and adults with sepsis (median angiopoietin-2 levels for respective controls levels are also shown for reference). **(B)** Correlations between HPSE activity and angiopoetin-2 levels at admission in both age groups. **(C)** Correlations between HPSE activity and change in serum albumin levels in both age groups. **(D)** Correlations between HPSE activity and maximum organ failure assessment scores within the first 24 h of hospitalization in both age groups. **(E)** Difference in HPSE activity between adult sepsis survivors and non-survivors (median HPSE activity in adult controls depicted for reference). Data presented as median (IQR). Comparisons made using the Wilcoxon rank-sum test, and correlations performed using Spearman’s rank correlation test.

Despite having lower circulating levels of HPSE activity, children with sepsis demonstrated more pronounced vascular endotheliopathy than adults, reflected by higher angiopoietin-2 levels at hospital admission and larger reductions in serum albumin over the first 24 h of hospitalization (Figure 2A). Notably, there were no significant differences in the amount of albumin [pediatric sepsis, median 0.3 g/kg (min-max 0–2.3) vs. adult sepsis, median 0 g/kg (min-max 0–3.6 g/kg), *p* = 0.129] or crystalloid resuscitation [pediatric sepsis, median 48 mL/kg (IQR 26, 82) vs. adult sepsis, 36 mL/kg (IQR 21, 79), *p* = 0.713] administered in the first 24 h of hospitalization between the pediatric and adult sepsis groups, suggesting that albumin infusions and/or crystalloid resuscitation did not significantly alter our findings. Organ failure scores followed a similar trend to biomarkers of endotheliopathy as children with sepsis exhibited higher organ failure scores than adults with sepsis (Figure 2A). In both age groups, plasma levels of HPSE activity demonstrated moderate correlations with angiopoietin-2 levels (Figure 2B), moderate inverse correlations with changes in serum albumin levels over the first 24 h of hospitalization (Figure 2C), and moderate correlations with organ failure scores in the first 24 h of hospitalization (Figure 2D). Adult non-survivors exhibited higher HPSE activity than survivors (Figure 2E) (mortality comparisons were not possible in children as no deaths occurred in the pediatric cohort, Table 1).

### 3.3 Candidate sources of circulating HPSE

**FIGURE 3:**
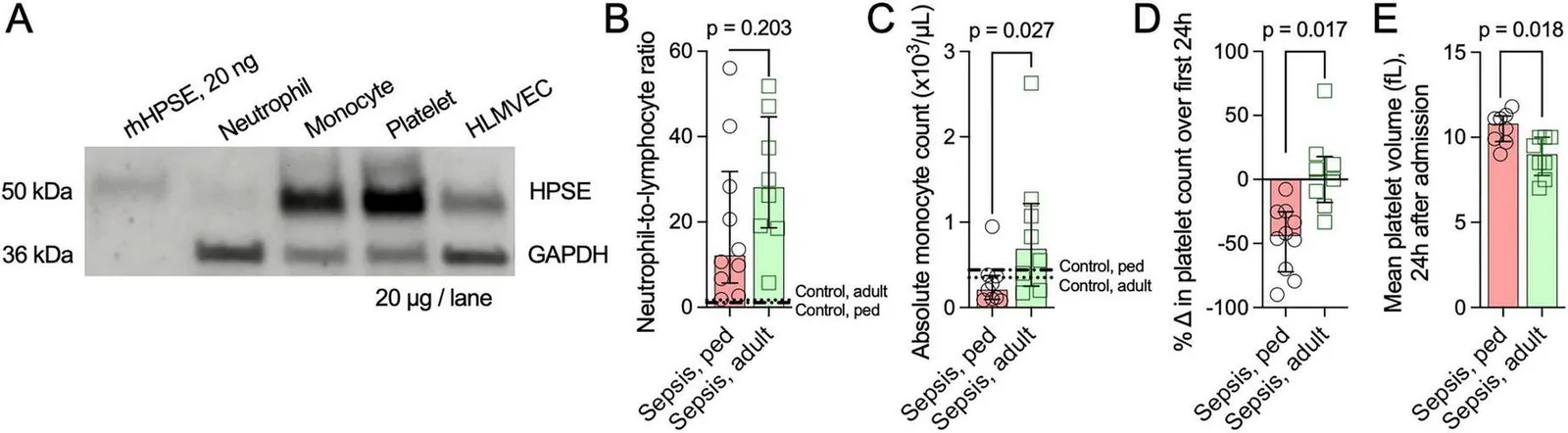
Candidate cellular sources of circulating HPSE during sepsis. **(A)** HPSE protein expression in neutrophils, monocytes, and platelets isolated from a peripheral blood specimen of a human adult male and primary human lung microvascular ECs (HLMVEC) relative to 20 ng recombinant, active human HPSE (cat#7570-GH-005, R&D Systems) with glyceraldehyde 3-phosphate dehydrogenase (GAPDH) serving as a loading control. **(B)** Ratios of absolute neutrophil count-to-absolute lymphocyte count, or neutrophil-to-lymphocyte ratio, at admission **(C)** absolute monocyte counts at admission, **(D)** change in circulating platelet counts over the first 24 h of hospitalization, and **(E)** mean platelet volumes 24 h after hospitalization for children and adults with sepsis with median values for respective controls depicted for reference. Data presented as median (IQR). Comparisons made using the Wilcoxon rank-sum test.

Prior work has shown that neutrophils, monocytes, platelets, and vascular ECs are the primary cell sources of HPSE in humans (24). Our own observations of HPSE levels in cell lysate of peripheral blood neutrophils, monocytes, and platelets from a healthy adult human, along with commercially available primary HLMVECs from a healthy adult human, suggest that platelets and monocytes may be the predominant generators of circulating HPSE (Figure 3A). Leveraging laboratory data available for subjects with sepsis, we evaluated whether differences in circulating cell counts may point to likely cell sources of HPSE within each age group. We observed that neutrophil-to-lymphocyte ratios (indicator of neutrophilic activation) were not significantly different between children and adults with sepsis while absolute monocyte counts (marker of monocyte proliferation) were significantly higher in adults with sepsis (Figures 3B, C). Change in platelet counts over the first 24 h of hospitalization and mean platelet volume 24 h after hospitalization (both suggestive of platelet activation, leading to consumption at the vascular endothelial surface and compensatory platelet generation by megakaryocytes) were significantly greater in children with sepsis (Figures 3D, E).

## 4 Discussion

In this focused study, we sought to determine age-related differences in circulating levels of HPSE activity and characterize the association of circulating HPSE activity with biochemical and clinical measures of endothelial injury and organ dysfunction in contemporary cohorts of children and adults with sepsis. We confirmed that plasma HPSE activity is elevated in children and adults with sepsis compared to controls, consistent with our prior observations in children and Schmidt et al.’s findings in adults (3, 4). HPSE activity correlated with biomarkers of endothelial dysfunction and organ injury in both age groups and was higher among adult non-survivors. However, contrary to our initial hypothesis, adults with sepsis demonstrated approximately 10-fold higher plasma HPSE activity than children despite the pediatric cohort developing more severe endotheliopathy and organ dysfunction, as reflected by higher angiopoietin-2 levels, greater reductions in serum albumin levels, and higher organ failure scores. The biochemical composition of circulating HS supported this age-dependent difference as the relative percentage of “cleavable” HS disaccharides was reduced in adults with sepsis but not in children, and inversely correlated with HPSE activity in adults, providing supportive biochemical evidence of greater HPSE-mediated glycocalyx degradation in the adult circulation. Together, these findings confirm HPSE-mediated glycocalyx degradation as a conserved feature of sepsis pathobiology across the age spectrum while revealing that the relationship between circulating HPSE activity and downstream endotheliopathy is not simply dose-dependent.

Several factors may explain the dissociation between lower circulating HPSE activity and more severe endotheliopathy in children with sepsis relative to adults. First, the pediatric endothelium may be inherently more susceptible to glycocalyx injury, such that even lower levels of HPSE activity produce disproportionate endothelial activation and barrier disruption. Our prior work has established that enzymatic removal of HS from the endothelial glycocalyx impairs shear stress-mediated AMPK/FoxO1 signaling, directly promoting angiopoietin-2 expression and secretion from endothelial cells (4). Angiopoietin-2 itself further amplifies glycocalyx breakdown through HPSE-dependent mechanisms (25). This feed-forward loop between glycocalyx degradation and angiopoietin-2-mediated endothelial destabilization may be particularly potent in the pediatric vasculature, where the endothelium lacks the chronic adaptive changes seen in aging adults. Second, the greater decline in platelet counts observed in children with sepsis suggests more pronounced platelet activation and endothelial adherence, which may contribute to endotheliopathy through platelet-dependent mechanisms independent of circulating HPSE levels. Third, the similar total circulating HS levels between pediatric and adult sepsis groups [children, 550 ng/mL (IQR 347, 1182) vs. adults, 449 (IQR 304, 1256), *p* > 0.99], despite markedly different HPSE activity, raise the possibility that non-HPSE sheddases–such as matrix metalloproteinases–contribute proportionally more to glycocalyx degradation in children, or alternatively that adults harbor lower baseline glycocalyx HS expression and thus a reduced abundance of HPSE substrate. Importantly, despite more pronounced acute endotheliopathy, no deaths occurred in the pediatric cohort, whereas adult non-survivors exhibited higher HPSE activity than survivors. This dissociation between endothelial injury severity and lethality further suggests that children may possess enhanced endothelial repair capacity, more robust glycocalyx regeneration, and/or differences in immune–vascular crosstalk that allow them to tolerate or recover from endothelial injury more effectively, whereas adults may be more susceptible to its downstream consequences.

The higher HPSE activity observed in adults may also reflect age-dependent differences in the cellular sources of circulating HPSE rather than a more intense overall inflammatory response. Our investigation of candidate cellular sources of circulating HPSE, while preliminary and hypothesis-generating, suggests that platelets and monocytes harbor the highest HPSE protein content among circulating cells and endothelial cells, consistent with prior reports (26–28). Moreover, prior transcriptomic analyses have demonstrated that children mount a more robust innate immune and inflammatory transcriptomic response to sepsis than adults, while adults exhibit diminished pathogen sensing and myeloid cell function, suggesting distinct cellular activation profiles between age groups (6, 29). We found that adults with sepsis exhibited significantly higher absolute monocyte counts, whereas children demonstrated evidence of greater platelet consumption. Therefore, monocyte proliferation may drive sustained HPSE release in the circulation of adults with sepsis, consistent with evidence that monocytes upregulate HPSE in inflammatory states (27, 30–32). However, in children with sepsis, platelet activation and subsequent endothelial adherence may sequester platelet-derived HPSE at the vascular surface rather than releasing it into the circulation, potentially explaining the lower plasma HPSE activity despite significant endothelial injury. This mechanism is supported by prior work demonstrating that thrombin selectively induces HPSE release from platelets via protease-activated receptor-1 and that platelet HPSE protein expression and activity are upregulated during clinical sepsis, with the active form of HPSE correlating with mortality.(26, 28) Though our preliminary findings require more thorough investigation, these age-dependent differences in HPSE cellular sourcing may have therapeutic implications, as interventions targeting HPSE inhibition may need to account for distinct cellular compartments of HPSE generation and release in children versus adults.

This study has several important limitations. The sample sizes are small, and the adult samples required pooling due to limited plasma volumes, which may have attenuated inter-individual variability and introduced averaging effects. The cross-sectional design captures a single, early time point and cannot delineate the temporal dynamics of HPSE activity, glycocalyx degradation, and endothelial activation over the course of sepsis. Readouts from the heparan sulfate disaccharide analysis should be viewed as supportive but not confirmatory evidence of HPSE enzymatic activity given that this analysis cannot provide disaccharide sequencing of circulating heparan sulfate chains and thus cannot provide detail on intact versus cleaved HPSE-binding domains along circulating heparan sulfate chains. The absence of pediatric mortality events precluded survival analyses in children. Additionally, the western blot analysis of HPSE cellular sources was performed using cells isolated from a healthy adult, and the relative HPSE content of these cell types during active sepsis–and in pediatric cells specifically–may differ. Future studies should employ larger, longitudinal cohorts with serial sampling to characterize the kinetics of HPSE activity across the age spectrum, incorporate single-cell and spatial transcriptomic approaches to define cell-specific HPSE expression during sepsis, and evaluate whether HPSE inhibition strategies require age-specific dosing or targeting to optimize therapeutic efficacy.

In summary, children and adults with sepsis exhibit elevated circulating HPSE activity that correlates with endothelial activation and organ dysfunction, confirming HPSE-mediated glycocalyx degradation as a conserved feature of sepsis pathobiology across the age spectrum. However, the magnitude and clinical implications of HPSE activity differ markedly by age: adults demonstrate substantially higher systemic HPSE activity driven by distinct cellular activation profiles–particularly neutrophilic and monocytic sources–with stronger associations with mortality, whereas pediatric sepsis is characterized by severe endotheliopathy despite comparatively lower circulating HPSE activity and no observed mortality, potentially reflecting heightened endothelial susceptibility to glycocalyx injury, platelet-mediated vascular sequestration of HPSE, and greater endothelial repair capacity. As sepsis is increasingly recognized as a heterogeneous syndrome, these age-dependent differences in HPSE biology, cellular sourcing, and vascular endothelial responses may prove to be important enriching variables for future prognostic models and underscore the need for age-stratified approaches to glycocalyx-directed therapeutic interventions.

## Data Availability

The raw data supporting the conclusions of this article will be made available by the authors, without undue reservation.
