# Supplementary material for: Age-related differences in circulating heparanase-1 activity in sepsis and correlation with systemic vascular endotheliopathy
Source: Front Med (Lausanne). 2026 Aug 12;13:1893157. doi: 10.3389/fmed.2026.1893157 (PMC13506940; doi:10.3389/fmed.2026.1893157)
Supplement: Supplementary file 1 [file Data_Sheet_1.pdf]

Supplementary Table 1. Statistical comparisons with and without outliers

Statistical comparison	Outlier	Statistical test	Spearman's rho value with outlier	Probability value with outlier	Spearman's rho value without outlier	Probability value without outlier
**Figure 1**						
HPSE activity, ped control vs ped sepsis	1 ped control	Wilcoxon rank-sum	n/a	0.013	n/a	0.002
Total HS level, ped control vs ped sepsis	0	Wilcoxon rank-sum	n/a	n/a	n/a	< 0.001
HPSE activity, adult control vs adult sepsis	1 ped control	Wilcoxon rank-sum	n/a	< 0.001	n/a	< 0.001
Total HS level, adult control vs adult sepsis	0	Wilcoxon rank-sum	n/a	n/a	n/a	< 0.001
HPSE activity vs HS, ped	1 ped control	Spearman's rank correlation	0.538	0.014	0.626	0.004
HPSE activity vs HS, adult	1 adult control	Spearman's rank correlation	0.901	< 0.001	0.900	< 0.001
Cleavable HS disaccharide	0	Kruskal-Wallis 1-way ANOVA	n/a	n/a	n/a	< 0.001
HPSE activity vs cleavable HS, adult	1 adult control	Spearman's rank correlation	-0.868	< 0.001	-0.865	< 0.001
**Figure 2**						
Angiopoietin-2 level, ped sepsis vs adult sepsis	1 adult sepsis	Wilcoxon rank-sum	n/a	< 0.001	n/a	< 0.001
Δ Albumin, ped sepsis vs adult sepsis	0	Wilcoxon rank-sum	n/a	n/a	n/a	0.041
Max SOFA, ped sepsis vs adult sepsis	0	Wilcoxon rank-sum	n/a	n/a	n/a	< 0.001
HPSE activity vs angiopoietin-2, ped	1 ped control	Spearman's rank correlation	0.549	0.012	0.668	0.002
HPSE activity vs angiopoietn-2, adult	1 adult control, 1 adult sepsis	Spearman's rank correlation	0.779	< 0.001	0.797	< 0.001
HPSE activity vs Δ albumin, ped	1 ped control	Spearman's rank correlation	-0.625	0.003	-0.727	< 0.001
HPSE activity vs Δ albumin, adult	1 adult control	Spearman's rank correlation	-0.591	0.010	-0.600	0.012
HPSE activity vs SOFA, ped	1 ped control	Spearman's rank correlation	0.405	0.076	0.500	0.029
HPSE activity vs SOFA, adult	1 adult control	Spearman's rank correlation	0.756	< 0.001	0.809	< 0.001
HPSE activity adult survivor vs adult death	0	Wilcoxon rank-sum	n/a	n/a	n/a	0.036
**Figure 3**						
NLR, ped sepsis vs adult sepsis	0	Wilcoxon rank-sum	n/a	n/a	n/a	0.203
AMC, ped sepsis vs adult sepsis	1 ped sepsis	Wilcoxon rank-sum	n/a	0.101	n/a	0.027
Δ Platelet count, ped sepsis vs adult sepsis	0	Wilcoxon rank-sum	n/a	n/a	n/a	0.017
MPV, peds sepsis vs adult sepsis	0	Wilcoxon rank-sum	n/a	n/a	n/a	0.018

AMC represents absolute monocyte count; ANOVA, analysis of variance; HPSE, heparanase; HS, heparan sulfate; MPV, mean platelet volume; NLR, neutrophil-to-lymphocyte ratio; SOFA, sequential organ failure assessment.
